# A Systematic Approach to Implementing, Evaluating, and Sustaining the Shared Citizenship Paradigm in the Disability Field

**DOI:** 10.3390/bs13120970

**Published:** 2023-11-25

**Authors:** Miguel Ángel Verdugo, Robert L. Schalock, Laura E. Gómez, Patricia Navas

**Affiliations:** 1 INICO, Universidad de Salamanca, 37005 Salamanca, Spain; verdugo@usal.es (M.Á.V.); patricianavas@usal.es (P.N.); 2 Hastings College, Hastings, NE 68901, USA; 3 Department of Psychology, Universidad de Oviedo, 33003 Oviedo, Spain

**Keywords:** shared citizenship paradigm, quality of life supports model, inclusion, CRPD, rights, empowerment, independent living, disability, quality of life, supports

## Abstract

The disability field continues to face challenges in transforming and implementing meaningful and effective changes in person-centered services and supports aligned with the principles of the Convention on the Rights of Persons with Disabilities. To guide this transformative process effectively, a paradigm must be operationalized through a systematic approach. This article outlines such a systematic approach, consisting of two components: (a) aligning the paradigm’s foundational pillars to the elements of an explanatory/implementation model (the Quality of Life and Supports Model) to facilitate the paradigm’s operationalization, acceptance, and application and (b) aligning implementation, evaluation, and sustainability strategies with ecological systems, implementation targets, and the paradigm’s foundational pillars to drive change across systems. The synergy and alignment between these components underscore the importance of linking public policies with professional and organizational practices to promote social inclusion and enhance the quality of life for people with disabilities. We provide practical examples highlighting the collaborative potential of this synergy and emphasize the significance of evidence-based and person-centered practices in promoting equal rights and social inclusion for people with disabilities.

## 1. Introduction

A paradigm is a collective and unifying set of values, assumptions, perceptions, and concepts that guide and frame the approach to a phenomenon. In the field of disability, a paradigm guides the development of policies and practices regarding the services and supports for people with disabilities and provides a framework not only for disability-related applications but also for inquiry and evaluation [1]. Historically, the disability field has been influenced by numerous paradigms. Chief among these have been paradigms that emphasize peculiarity, abnormality, defects, the need for institutionalization and segregation, the object of dread or pity, disease, or eternal childhood [2]. A paradigm shift occurs when there are changed perceptions and actions regarding how things should be thought about, done, or made [3,4,5,6]. As a result of the shift, a new paradigm emerges that reflects new or different values, assumptions, perceptions, and concepts that are perceived to overcome many of the inadequacies or weaknesses of the preceding paradigm(s).

Over the last 3–4 decades, the disability field has experienced a number of shifts and witnessed the emergence of new paradigms that have focused on community living and inclusion [7,8,9,10,11,12], personal autonomy [5,13], human and legal rights [14,15,16,17,18,19], and human capacity [20,21,22]. These paradigms have typically emphasized a specific area and conceptual issues (e.g., community living, integrated employment, inclusive education, capacity, or rights) rather than describing a systematic approach to the paradigm’s implementation, evaluation, and sustainability.

Due to the significant transformation in policies and practices concerning people with disabilities, the disability field has recently experienced the emergence of a new, more all-encompassing paradigm that offers a comprehensive framework for application, inquiry, and evaluation. This new paradigm is known as the Shared Citizenship Paradigm (SCP) [1,2]. The SCP is increasingly providing a framework for disability-related policies and practices for people with disabilities by its focus and emphasis on their active engagement and full participation as equal, respected, valued, participating, and contributing members in all aspects of society [2].

The SCP, which represents a paradigm shift from those single-focused paradigms mentioned earlier, is driven by two primary forces: (a) the widespread adoption of civil and human-rights conventions, which emphasize the rights and equal treatment of individuals with disabilities; and (b) the ongoing transformation taking place in the disability field, where there is a growing recognition of the need for inclusive practices, empowering approaches, and evidence-based practices.

Regarding the first force, the exercise of individual rights, like any other citizen, enables people with disabilities to achieve satisfaction, wellbeing, and quality of life. Civil and human-rights conventions advocate for the transformation of organizations and systems to ensure the adoption of public policies and system-delivery practices that embody disability-rights principles (e.g., equity, inclusion, self-determination, and empowerment), self and family advocacy, the implementation of the supports model for person-centered planning, and the formation of partnerships involving consumers, support providers, professionals, organizations, and societal members [4,23,24,25].

Concerning the second force, the ongoing transformation in the disability field is characterized by several key aspects [26]: (a) using accurate terminology and the right words; (b) adopting a functional and holistic approach that considers person’s strengths and contextual factors rather than exclusively focusing on persons’ limitations; (c) embracing the supports model and evidence-based practices to enable people with disabilities to live as independently as possible, according to their values and personal goals; (d) implementing outcome evaluation and assessing the impact of interventions and support services on individuals’ quality of life and functioning; (e) empowering people with disabilities and families through knowledge, tools, and autonomy to make decisions about their lives; (f) recognizing the multidimensional properties of context and how they interact and affect people’ lives; and (g) emphasizing professional responsibility and ethical obligations to provide quality support and advocacy for people with disabilities.

Consistent with both civil and human-rights conventions and the characteristics of the current transformation in the field of disability [5,13,14,15,20], the SCP serves as a guiding framework for transforming and sustaining best-practice services and supports for people with disabilities. This is because the SPC and its foundational pillars (a) are consistent with the principles and frameworks outlined in civil and human-rights conventions; (b) incorporate an updated and contemporary set of values and beliefs about people with disabilities, emphasizing their right to full participation in all aspects of life and society; (c) move the field beyond a general focus on environmental factors and instead address specific contextual factors that influence the manifestation of disability, and ameliorate the barriers that hinder the achievement of shared citizenship; and (d) incorporate the exponential growth in knowledge about the causes, characteristics, and amelioration of disability.

To be sustainable, a paradigm’s foundational pillars need to be supported through advocacy, research, policies, practices, resources, and consumer involvement [3,6,27,28]. The foundational pillars that shape the SCP are (1) a holistic approach to etiology and amelioration of disability; (2) a contextual model of human functioning; (3) disability-rights principles; and (4) person-centered planning and evaluation.

1. Holistic approach to etiology and the amelioration of disability. Scientific knowledge has allowed us to develop different approaches to understanding people with disabilities and how to support them. As described by Schalock et al. [29], a holistic approach to disability encompasses four perspectives: biomedical, psychoeducational, sociocultural, and justice. Each of these four perspectives has both its own explanation of the etiology or risk factors associated with disability and specific interventions or supports for its amelioration. One’s shared citizenship status can be influenced by one or more etiological or amelioration factors associated with these four perspectives;
2. The contextual model of human functioning encompasses context-based sociocultural factors that affect (positively or negatively) the manifestation and amelioration of disabilities. As an independent variable, for example, the context includes personal and environmental characteristics that are not typically manipulated, such as age, language, culture and ethnicity, sex, and family. As an intervening variable, context includes organizations, systems, supports, and societal policies and practices that can be manipulated to enhance human functioning and personal outcomes [16,30,31];
3. Disability-rights principles, including equity, inclusion, self-determination, and empowerment are a prerequisite for shared citizenship [4,17,21,22,32,33,34];
4. Person-centered planning and evaluation that provides value to the person, involves the meaningful participation of people with disabilities, and incorporates knowledge of best practices related to the provision and evaluation of individualized services and supports [9,35,36,37,38]. Specifically, person-centered planning and evaluation (a) is a systematic endeavor that aligns an understanding of the person’s support needs, the provision of individualized systems of support, and the evaluation of valued personal outcomes; (b) involves a collaborative partnership between the individual and a support or service provider; and (c) incorporates the knowledge, skills, and resources of the partnership to assess support needs, plan and implement systems of supports, and evaluate personal outcomes [39].

Regrettably, the full engagement and participation of people with disabilities as equal, respected, valued, participating, and contributing members of all aspects of society is still not a reality for many. While there have been positive strides towards this goal in some service-delivery systems [10,40,41], the disability field continues to face challenges in transforming and implementing meaningful and effective changes in person-centered services and supports that are consistent with the principles of the Convention on the Rights of Persons with Disabilities (CRPD) [25].

Hence, to guide and frame this transformative process effectively, a paradigm must be operationalized through a systematic approach. The primary goal of this article is to delineate such a systematic approach for implementing, evaluating, and ensuring the long-term sustainability of the SCP within the disability field. This systematic approach underscores the utmost importance of establishing strong connections between public policies and evidence-based, person-centered professional practices to promote equal rights, social inclusion, and quality of life for people with disabilities. It is crucial to note that, while this systematic approach finds its roots primarily in advancements and contributions from the intellectual and developmental disabilities field, the framework we propose is equally applicable to all types of disability (i.e., physical, sensory, cognitive, or psychosocial disabilities) and levels of support needs (i.e., intermittent, limited, extensive, or pervasive).

## 2. Overview of the Systematic Approach

The systematic approach described in this article involves two components: (a) aligning the SCP’s foundational pillars to elements of an explanatory/implementation model to facilitate the paradigm’s operationalization, acceptance, and application; and (b) aligning implementation, evaluation, and sustainability strategies to ecological systems, implementation targets, and the paradigm’s foundational pillars to bring about change across ecological systems.

### 2.1. Aligning the SCP and an Explanatory/Implementation Model

The first component involves aligning the SCP’s foundational pillars to elements of a currently used explanatory/implementation model. The importance of this first component is that (a) a paradigm needs an operationalized and measurable model to be more easily implemented, evaluated, and sustained; and (b) an explanatory/implementation model needs a paradigm to give it context, relevance, credibility, and content [2,20].

Over the last three decades, two powerful forces have come together into an explanatory/implementation model that is aligned with the SCP’s definition and foundational pillars. These two forces are the quality of life (QOL) concept and the concept of individualized supports. When joined together, these two powerful forces form the Quality of Life Supports Model (QOLSM) [3,42,43]. The concept of QOL provides a framework for policy development, best practices, and outcome evaluation. This is because QOL is characterized by [4] (a) value-based principles that involve its multidimensional and contextual nature, encompassing both subjective (personal feelings, perceptions, and experiences) and objective components (measurable indicators), and enriched by factors such as self- determination, access to resources, a sense of purpose in life, and a feeling of belonging, and (b) its universal nature. It is a universal concept with the same domains for all people, but it places a strong emphasis on the individual and on achieving valued person-referenced outcomes (the relative value of QOL domains varies among individuals). Individualized supports serve as a framework for planning and delivering a coordinated set of person-centered strategies that prevent or mitigate the impact of disability, promote personal development and interests, and enhance overall functioning and wellbeing.

The QOLSM contributes both theoretically and practically to the disabilities field. Theoretically, the model integrates three critical catalysts that bring about positive change in peoples’ lives: connections, interactions, and facilitating conditions. The connections a person or a family has with other people, social networks, and technology provide the opportunities to enhance a person’s or family’s wellbeing and QOL. The interactions that result from these connections provide the systems of support that facilitate functioning, interests, and wellbeing. The facilitating conditions that are basic to both the connections and interactions focus on principle-based opportunity development and value-based support provision. Practically, the QOLSM integrates the significant characteristics of the current transformation in the disabilities field [42,43].

The alignment of the foundational pillars of the SCP and the elements of the QOLSM facilitates the paradigm’s operationalization, acceptance, and application (Table 1).

### 2.2. Aligning Ecological Systems, Implementation Targets, and SCP Foundational Pillars

The second component involves aligning implementation, evaluation, and sustainability strategies to ecological systems, implementation targets, and the paradigm’s foundational pillars (see Table 2). The importance of this second component lies in applying the SCP across ecological systems (i.e., macro-, meso-, and microsystem) [44]. This allows us to address the contextual factors that impact a person’s life, thereby incorporating a holistic and culturally and contextually relevant approach to providing individualized support, facilitating organizational transformation, and driving systems change [4,25].

Indeed, for a paradigm like the SCP to be effective and drive meaningful change, it is crucial to operationalize and apply it across the three nested ecological systems that impact human functioning. Bronfenbrenner’s [44] ecological theory is often used to operationalize and address the contextual factors that impact a person’s life, thereby providing a holistic culturally and contextually relevant approach to provide individualized support, facilitate organizational transformation, and bring about systems change. Thus, Bronfenbrenner’s ecological systems theory provides a comprehensive framework for understanding the various interrelated systems that impact human functioning, with the individual at the core. In this model, the individual’s experiences and development are influenced by multiple concentric circles of systems that radiate outward from the individual. In this context, at the microsystem level, contextual factors include the individual’s immediate social setting, family, close friends, and colleagues [2]. At the mesosystem level, contextual factors include the neighborhood, community, and the organization or service providing support [2]. At the macrosystem level, contextual factors include the larger service delivery system and the overarching patterns of culture, society, and sociopolitical influences [2]. Finally, the chronosystem highlights the dynamic nature and recognizes that these various systems are not static; they change and interact over time. Events and transitions in an individual’s life, as well as historical and societal changes, can have a significant impact on a person’s development.

In this sense, the SCP is impacting disability-related services and support systems on three levels: macro-, meso-, and microsystem [7,8,45,46]. At the macrosystem level, there have been significant changes in public policies that incorporate disability rights, emphasizing (a) inclusion (ensuring that individuals with disabilities are fully integrated into society and have equal access to education, employment, public services, and community participation); (b) equity (distributing resources and opportunities in a way that addresses their specific needs); (c) self-determination (giving them control over their own lives, choices, decisions, and life directions); and (d) empowerment (enabling them to have the knowledge, skills, and resources to advocate for their rights). At the mesosystem level, service and support organizations are undergoing transformations by adopting a focus on person-centered approaches to planning and evaluation. They are increasingly providing individualized supports that align the needed supports with the necessary systems of supports and the desired personal and valued outcomes of people with disabilities. At the microsystem level, people with disabilities are experiencing greater opportunities to live, work, and receive education in inclusive environments. Moreover, they are actively participating and becoming more involved in the development, implementation, and evaluation of their individual support plans, further empowering their decision making and autonomy [7].

**Figure 1:**
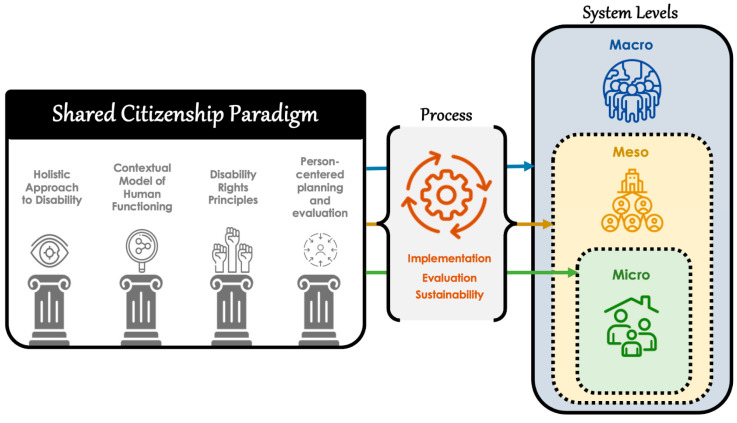
Components of a systematic approach to implementing and sustaining the SCP.

Based on the considerable literature, meaningful and sustainable organization transformation and systems change requires both a paradigm to guide the transformation and change, and a systematic approach to bring about and sustain the desired transformation and change [6,27,28]. To address these two requirements, in Figure 1 we (a) illustrate how to integrate the four pillars of the SCP with specific implementation strategies at the systems level; (b) exemplify how the four pillars of the SCP can be operationalized, evaluated and implemented across the micro-, meso-, and macrosystems; and (c) highlight how the sustainability of the paradigm can be enhanced by supporting its four foundational pillars through an explanatory/implementation model such as the QOLSM. Figure 1 also illustrates (a) the interconnections and interdependence among the SCP, the ecological systems, and the processes of implementation, evaluation, and sustainability of the SCP; and (b) the dynamic and holistic approach to advancing the rights and inclusion of people with disabilities, with the ultimate goal of fostering a society that embraces shared citizenship for all.

## 3. Achieving Change through Applying the SCP and the QOLSM

The SCP serves as a valuable and comprehensive framework for evaluating, implementing, and researching inclusive evidence-based practices in the field of disabilities. Given its emphasis on rights and self-determination, this paradigm provides a contextual lens through which to examine and advance public policies and professional and organizational practices. The QOLSM has recently emerged as a significant application model to not only operationalize and implement the SCP but also to facilitate organizational and systems change [3,42,43]. This is due in part to the synergistic interaction between the SCP and the QOLSM, leading to a more holistic and impactful approach to supporting individuals with disabilities, and fostering greater social inclusion and overall wellbeing.

In this context, the QOLSM is serving as a powerful instrumental model for [3,42,43] (a) policy development and system change at the macrosystem level, guiding the creation of inclusive policies that prioritize and promote the QOL of people with disabilities through the provision of supports and services tailored to meet their unique needs and aspirations; (b) facilitating organizational transformation at the mesosystem level, by implementing person-centered practices that empower people with disabilities and allow them to actively shape their support systems and life choices, together with practices aimed at continuous improvement of service quality through the evaluation and monitoring of the effectiveness of the supports received; and (c) outcome evaluation at the microsystem level, providing a robust model for assessing the impact of individualized supports plans on the QOL of people with disabilities (i.e., validating evidence-based practices and refining them continuously).

Both the SCP and the QOLSM place significant emphasis on the relationship between public policies and professional and organizational practices to advance the rights of people with disabilities. Both also share the goal of promoting equal rights and social inclusion. However, the QOLSM, being an integrated model within the SCP, plays a pivotal role as the best catalyst in translating policies and conceptual principles into meaningful inclusive person-centered practices that achieve real and measurable advances. It bridges the gap between theory and implementation, ensuring that the principles outlined in public policies are effectively translated into real and measurable advances in the lives of people with disabilities. By emphasizing QOL and individualized supports, the QOLSM facilitates the development of services and supports that are tailored to peoples’ specific needs and aspirations, ensuring their rights are upheld and respected [3,42,43].

Through this linkage and alignment (as depicted in Table 1), the SCP and QOLSM collaborate to create a seamless flow of influence from public policies to practical implementations. The synergistic link leads to a more cohesive and effective approach to advancing the rights and wellbeing of people with disabilities. It not only underscores the importance of policy development but also highlights the critical role of evidence-based and person-centered practices in achieving meaningful advancements. Next, we provide some illustrative examples of practical implementations showcasing this synergy between the SCP and the QOLSM. These examples highlight its collaborative potential in effecting meaningful advancements.

A prime example is the development and use of the #Rights4MeToo scale [43,47] at the three-system level. The scale is an assessment tool specifically designed to evaluate the rights outlined in the CRPD [25] and to operationalize the QOLSM by quantitatively measuring many of the concepts delineated in the model (QOL domains; CRPD articles; knowledge, supports, barriers, and opportunities to exercise rights). The #Rights4MeToo scale is a tool designed to serve various important purposes [43,47]:

1. Empowering people (i.e., the microsystem): one of its primary goals is to provide accessible means and opportunities for people with disabilities, especially those with intellectual and developmental disabilities, to identify and express their needs regarding their rights and daily situations where they face discrimination or situations that do not comply with what has been ratified in the CRPD;
2. Supports improvement and program evaluation (i.e., mesosystem): The scale can also be used by professionals and family members to assess and identify the strengths and weaknesses of individuals in relation to their rights. By doing so, they will better tailor their assistance and support for people with disabilities. Additionally, the scale can be employed to evaluate and monitor the effectiveness of the programs and supports implemented by organizations in terms of ensuring and promoting the rights of people with disabilities;
3. Policy guidance (i.e., macrosystem): It can serve as a guide for assessing and monitoring public policies and their alignment with the rights and needs of individuals with disabilities. The scale helps pinpoint areas of concern or gaps in rights implementation, measure the impact of policy changes and initiatives, set priorities for policy development and reform, and monitor policy compliance.

Another notable example of public-policy implementation at the macrosystem level, aimed at promoting the rights and inclusive opportunities for all people with disabilities, can be observed in the deinstitutionalization strategy in Europe [23]. This initiative encompasses a wide range of population groups, including people with disabilities, homeless people, children, adolescents, adults, older adults, and other institutionalized groups. The strategy is founded on the premise of creating alternatives to residential institutions and segregated care settings and thus facilitating the development of community-based services [8,11,12,48,49]. By placing individual life projects at the forefront, this approach aims to transform the support and care model. European states and social organizations are actively implementing significant changes within the care/rehabilitation system, with a focus on fostering greater independence, self-determination, and social participation for individuals. Aligned with the European guidelines [23], the Spanish Strategy on Disability 2022–2030 [50] also endeavors to advance the human rights of people with disabilities and their families through governmental administrations [19,51,52,53]. This strategy is developed through a process of civil dialogue and in collaboration with representative entities for people with disabilities and their families.

In line with this strategy, Component 22 of the Government’s Recovery, Transformation, and Resilience Plan, supported by Next Generation EU Funds, aims to develop a “Shock Plan for the Care Economy and Reinforcement of Inclusion Policies”. The Ministry of Social Rights and the 2030 Agenda, recognizing the need to shift from an institutionalized support model to a rights-based approach, has allocated 110 million euros to fund 20 pilot projects focused on innovating the provision of supports to individuals at risk of institutionalization and social exclusion. One of the notable pilot projects is ‘My Home: A Life in the Community’, led by the Spanish organization Plena inclusión, which involves more than 900 service providers supporting people with intellectual and developmental disabilities. This project serves as a clear example of the necessary changes required at all system levels and how the SCP and QOLSM play a pivotal role in its implementation. At the macrosystem level, there is a clear commitment to facilitate the development of new support methods aligned with scientific evidence on deinstitutionalization and in line with the CRPD. At the mesosystem level, the principles of QOLSM are key in effecting changes through various pathways, including (a) the implementation of person-centered plans that empower individuals to make decisions about where, how, and with whom to live; (b) the development of new roles, such as community connectors and facilitators, who work with people with disabilities to foster community engagement; and (c) the training of professionals to provide supports according to a rights-based approach, including active support, person-centered planning, support decision making, and positive behavior support, thereby promoting a cultural shift within organizations. At the microsystem, people with disabilities, along with their families or circle of supporters, play a key role in the decision-making process. With the necessary supports, they are empowered to choose where, how, and with whom they want to live, regardless of their support-needs intensity (more than 60% have high support needs). This project not only seeks innovation but also the development of evidence-based practices.

Based on previous experiences [7], the Institute for Community Integration (INICO) at the University of Salamanca actively participates in the project evaluation through a longitudinal study (data are collected before the transition to houses in the community, 9 and 18 months after the move). The evaluation focuses on personal outcomes, such as QOL domain scores, participation measures, and decision-making indicators, comparing them to those of a similar-sized sample that has chosen to remain in their current residential facility. Additionally, the evaluation aims to analyze possible changes in families’ QOL, organizational culture, and professional practices. To date, its results show a clearly positive trend towards the improvement of participants’ functioning and quality of life.

As previously mentioned, social organizations and professionals play a pivotal role in the implementation of public policies, as together they are responsible for translating plans and daily activities into transformative services for people with disabilities and their families. Therefore, it is essential to emphasize that all planning and evaluation activities should be person centered at the mesosystem and microsystem levels [54,55]. Thus, professionals and organizations need to adopt a community-based, nondiscriminatory, and empowering approach [56]. Embracing this more conducive perspective ensures the exercise of rights and empowers individuals to take control over their own lives. It is crucial to provide professionals and organization/system staff with up-to-date training on the most current approaches and the most effective and efficient practices and processes of organizational transformation [57,58,59,60,61,62,63]. In this sense, the postgraduate training programs of universities are very important. Noteworthy examples are the three master’s programs offered for over 30 years by INICO at the University of Salamanca. These programs have trained more than 1,000 professionals from various organizations in Spain and Latin America, fostering a paradigm and model that is centered on assessing and improving the QOL of people with disabilities through an individualized support approach (i.e., the SCP and the QOLSM). Most of the organizations in favor of people with disabilities in Spain emphasize as their mission both the improvement of the QOL of their members, and that of their families, and the promotion of the exercise of rights, aligning themselves with the QOLSM and the SCP.

## 4. Conclusions

We have introduced an innovative and timely approach with broad applicability across all types of disabilities (i.e., sensory, physical, cognitive, and psychosocial). This distinctive alignment between the SCP and the QOLSM presents a practical framework for achieving significant positive changes in the lives of individuals with disabilities. It empowers them to enjoy more equitable and inclusive lives. By seamlessly connecting public policies with professional and organizational practices, this approach effectively tackles the central challenge confronting practitioners and researchers in the field. In the contemporary landscape, characterized by evolving modes of institutionalization, this approach assumes heightened significance as it harmonizes with the urgent imperative to deliver personalized supports and embrace holistic, person-centered solutions for individuals with disabilities.

We have emphasized that the field of disability is challenged nationally and internationally to bring about meaningful changes in policies, services, and supports for people with disabilities. To address this challenge, we discussed how meaningful change in organizations and systems requires both a paradigm to guide the change and a systematic approach to bring about and sustain the change. To that end, we described the SCP and its four foundational pillars and their potential to facilitate the paradigm’s operationalization, acceptance, and application.

Throughout the article, we have also described and emphasized the importance of the components of a systematic approach to implementing, evaluating, and sustaining the emerging SCP. As depicted in Figure 1, these involve (a) operationalizing the paradigm’s four foundational pillars; (b) using literature-based strategies to implement, evaluate, and sustain the paradigm through advocacy efforts, policies and practices, allocation of resources, and meaningful consumer involvement (Table 2); and (c) applying the SCP across the macrosystem, mesosystem, and microsystem. Our intention was to present a comprehensive theoretical framework and systematic approach for implementing, evaluating, and ensuring the long-term sustainability of the SCP within the disability field. Our current contribution lays the theoretical foundation and provides a structured approach for implementing the paradigm, with the hope that it will guide and inspire further empirical investigations to demonstrate its effectiveness and validation, and we encourage researchers in the field to undertake such endeavors.

Despite the specificity of the strategies and techniques discussed in this article, we need to acknowledge three fundamental principles of change. First, change takes time. We recognize the complexity of change, the frequent resistance to change, and that organizations and systems are at different levels of development due to macro- and mesosystem factors. In this regard, the specific strategies presented in Table 2 should provide a guide for initiating change and learning from others. Second, change involves a collaborative process. Our experience indicates clearly that the successful implementation of either all or parts of the SCP requires planning and collaborative work among policymakers, service/support providers, people with disabilities and their families, professionals, and researchers, as together they work to bring about meaningful change in the policies, services, and supports to people with disabilities [63,64,65]. Third, change is facilitated through a systematic approach and process such as that described in this article. Our hope is that this systematic approach to implementing and sustaining the SCP will both reduce the time required to make significant changes in policies and practices regarding the services and supports provided to people with disabilities, and facilitate the partnerships and collaborative efforts to bring such change about.

## Figures and Tables

**Table 1:** Alignment of the SCP’s foundational pillars and the elements of the QOLSM.

SCP Foundational Pillars	QOLSM Elements
1. Holistic Approach to Disability: A. Biomedical Perspective B. Psychoeducational Perspective C. Sociocultural Perspective D. Justice Perspective	QOL Domains: A. Physical and Emotional Wellbeing B. Personal Development and Material Wellbeing C. Social Inclusion and Interpersonal Relationships D. Rights and Self-Determination
2. Contextual Model of Human Functioning	Alignment of personal goals with context-based support needs, support strategies, and valued QOL outcomes
3. Disability-Rights Principles	QOL principles that recognize the human and legal rights of people with disabilities, as enshrined in the CRPD ^1^. These principles and rights embody equity, inclusion, self-determination, and empowerment
4. Person-Centered Planning and Evaluation	− Individual support needs aligned with individualized support strategies and valued outcomes − Individual Supports Plan − Person-centered QOL domain-referenced outcome evaluation

^1^ CRPD: Convention on the Rights of Persons with Disabilities.

**Table 2:** Alignment of strategies to ecological systems, implementation targets, and SCP foundational pillars.

System Level	Primary Implementation Role	Paradigmatic Pillar(s) Addressed	Exemplary Implementation, Evaluation, and Sustainability Strategies	Impact
Micro	Involving the person in developing, implementing, and evaluating person-centered services and supports	1. Person-centered planning and evaluation	Implement person-centered planning activities that involve the person in developing and implementing his/her individual support plan Implement person-centered evaluation activities that involve the person in evaluating his/her status on QOL domains and/or shared citizenship indicators Provide training to professionals and direct support staff regarding (a) the principles and methods of person-centered planning and evaluation and (b) the four foundational pillars of the shared citizenship paradigm Develop innovation in plans and actions and implement evidence-based practices	Personal growth and development Increased equity, self-determination, inclusion, and empowerment Engagement and full participation in society
Meso	Implementing best practices	1. Holistic approach to etiology and amelioration 2. Disability-rights principles 3. Person-centered planning and evaluation	Acknowledge in the organization’s mission statement and policies the multidimensionality of the causes of disability and the various types of supports (i.e., systems of supports that involve personal control and autonomy, inclusive environments, generic supports, and specialized supports) that can be used to ameliorate a person’s functional limitations Implement best practice policies and practices Conduct contextual analysis to identify context-based factors that facilitate or inhibit human growth and personal development Operationalize the four SCP foundational pillars and develop support systems to sustain their ongoing use and influence in guiding organization/system improvement Implement the QOLSM Build organization and system capacity to bring about meaningful change through workforce development, technology, partnerships, harnessing the power of licensure, community involvement, and multiple types of resources (e.g., expertise, experience, social and financial capital, moral and legal capacity, and efficient use of time) Develop and implement individual support teams that (a) align assessed support needs, individualized systems of supports, and valued outcomes; and (b) develop opportunities for shared citizenship Develop innovation in services and supports and implement evidence-based practices	Organization transformation Sustainable best practices
Macro	Developing policies	1. A holistic approach to etiology and amelioration 2. Contextual model of human functioning 3. Disability-rights principles 4. Person-centered planning and evaluation	Incorporate into disability policies: (a) the multidimensionality of human functioning that involves the four perspectives on disability (biomedical, psychoeducational, sociocultural, and justice) and (b) the four pillars of the SCP (holistic approach to disability, a contextual model of human functioning, disability-rights principles, and person-centered planning and evaluation) Develop policies that encourage and promote human and legal rights (e.g., community living, work, education, self-advocacy, and supported decision making) State measurable indicators of shared citizenship, such as the core QOL domains of personal development, self-determination, interpersonal relations, social inclusion, rights, emotional wellbeing, physical wellbeing, and material wellbeing Develop policies that mandate: (a) person-centered planning and evaluation and (b) monitoring the status, impact, and sustainability of the SCP Policies including subjective and objective measures to evaluate results	Cultural and societal change Sustainable public policy

## Data Availability

No new data were created or analyzed in this study. Data sharing is not applicable to this article.
